# Supplementary figures and images for: The stoichiometry of minor-to-major pilins regulates the dynamic activity of the type IVa competence pilus in Vibrio cholerae
Source: PLoS Genet. 2026 Jun 4;22(6):e1012188. doi: 10.1371/journal.pgen.1012188 (PMC13249403; doi:10.1371/journal.pgen.1012188)

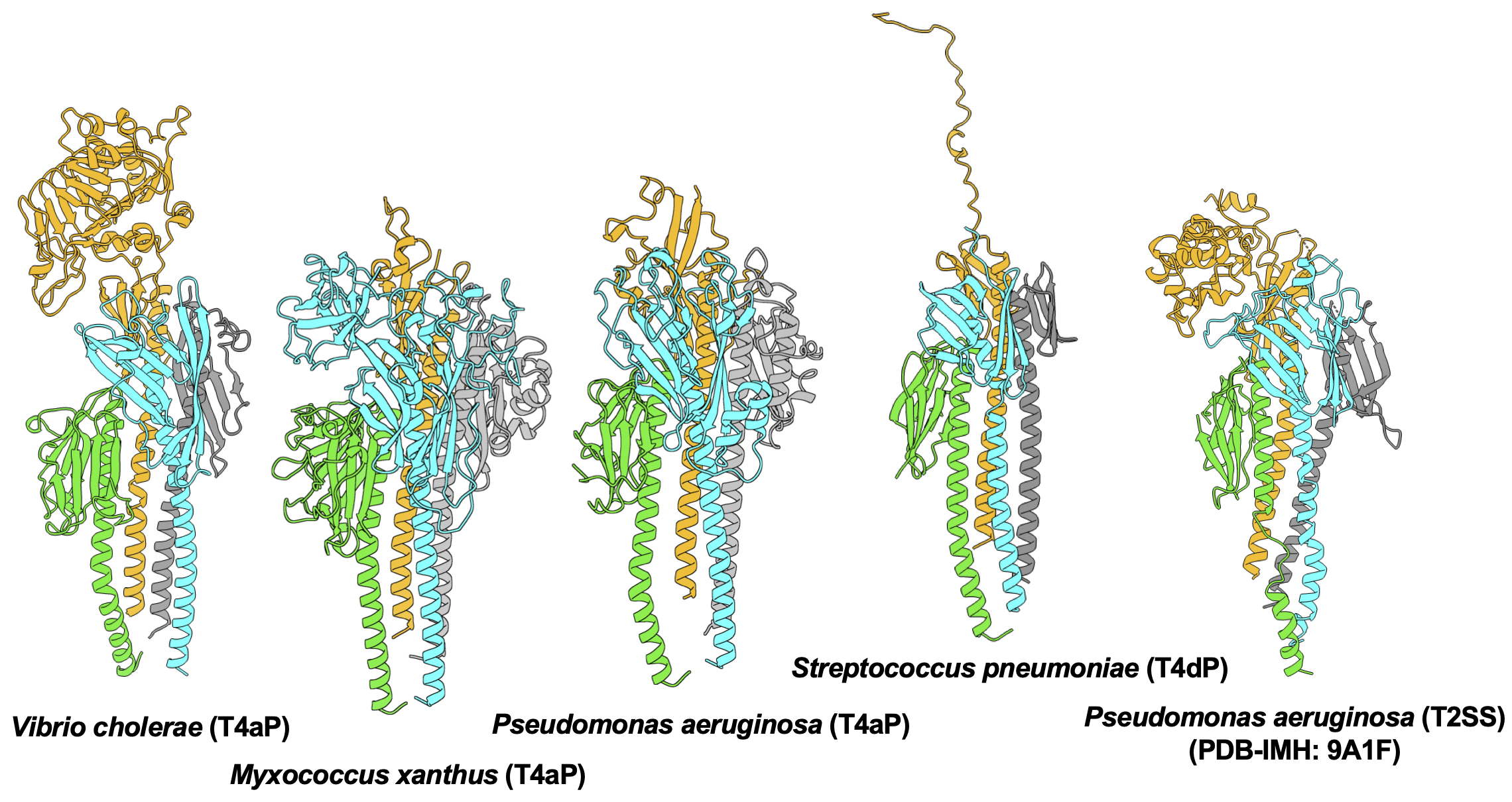

Supplement: S1 Fig — AlphaFold-multimer models of the core minor pilins from V. cholerae (T4aP), M. xanthus (T4aP; Treuner-Lange, Chang et al. 2020), P. aeruginosa (T4aP; Nguyen, Sugiman-Marangos et al. 2015), S. pneumoniae (T4dP; Christman and Dalia 2025), and P. aeruginosa (T2SS; Escobar et al., 2021). Pilins are colored based on their structure and position within the predicted complex. Green = FimU/T, Cyan = PilW, Grey = PilV, Yellow = PilX. (TIFF) [file pgen.1012188.s001.tiff]

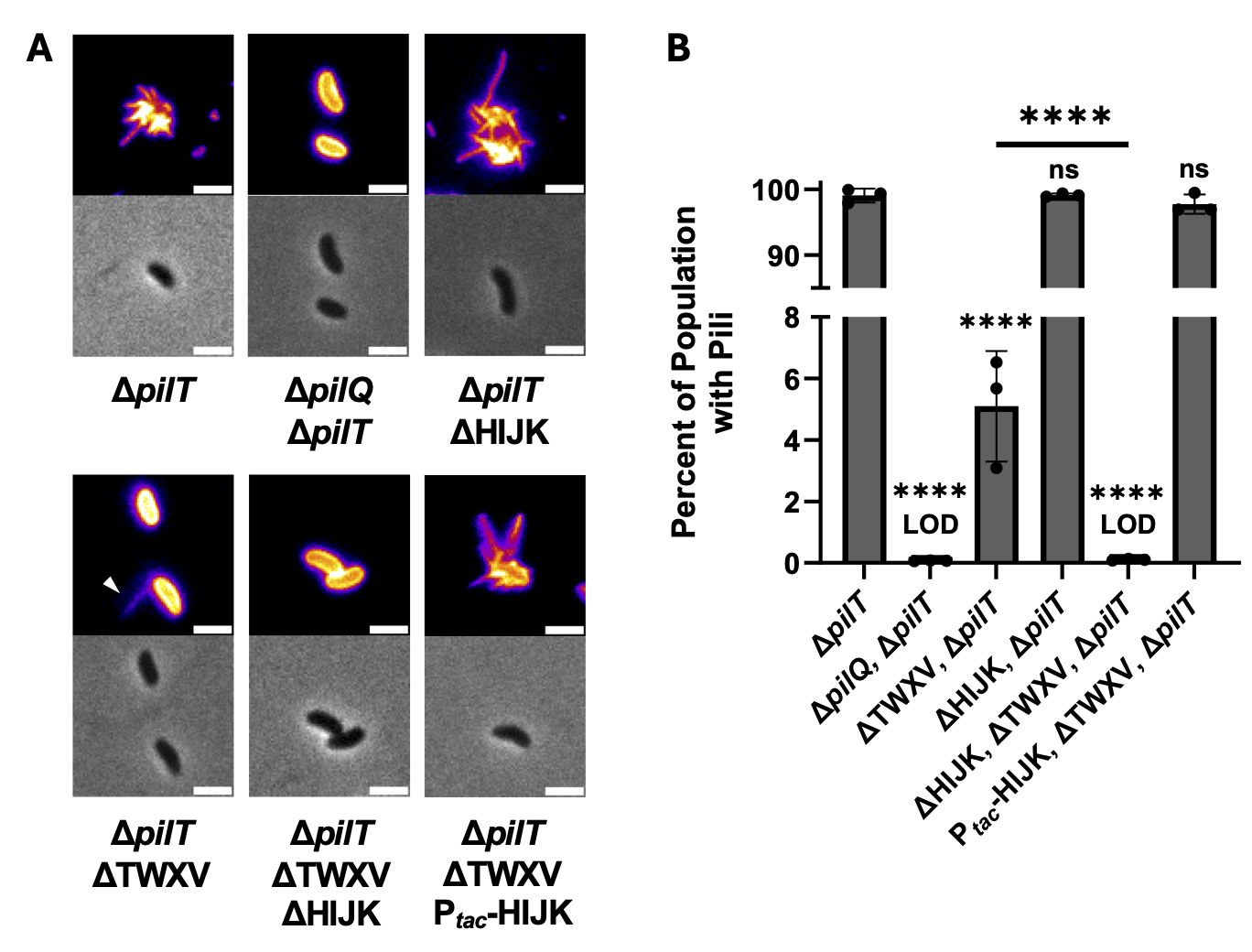

Supplement: S2 Fig — (A) Representative images of the indicated strains stained with AF488-mal. All strains were induced with 100 µM IPTG. Images are false colored with the “Fire” LUT in Fiji to make rare pili easier to see (white arrows). Scale bar, 2 µm. (B) Quantification of surface piliation in the indicated strains stained with AF488-mal, as depicted in A. For these assays, the parent strain is ∆vesC ∆epsM. The ∆vesC mutation suppresses the lethality typically associated with loss of T2SS activity in V. cholerae, and the ∆epsM mutation disrupts an essential T2SS machine component to ensure that all strains lack T2SS activity. Data in B are from 3 independent biological replicates and shown as the mean ± SD. The number of cells quantified varied for each strain and replicate. The total number of cells analyzed for each strain: ΔpilT = 3067, ΔpilT ΔpilQ = 4523, ΔpilT ΔHIJK = 3123, ΔpilT ΔTWXV = 4273, ΔpilT ΔTWXV ΔHIJK = 3131, ΔpilT ΔTWXV Ptac-HIJK = 2647. Statistical comparisons were made by one-way ANOVA with Tukey’s multiple comparison test of the log-transformed data. NS, no significance; **** = p < 0.0001. LOD, limit of detection. Statistical identifiers directly above bars represent comparisons to ∆pilT. (TIFF) [file pgen.1012188.s002.tiff]

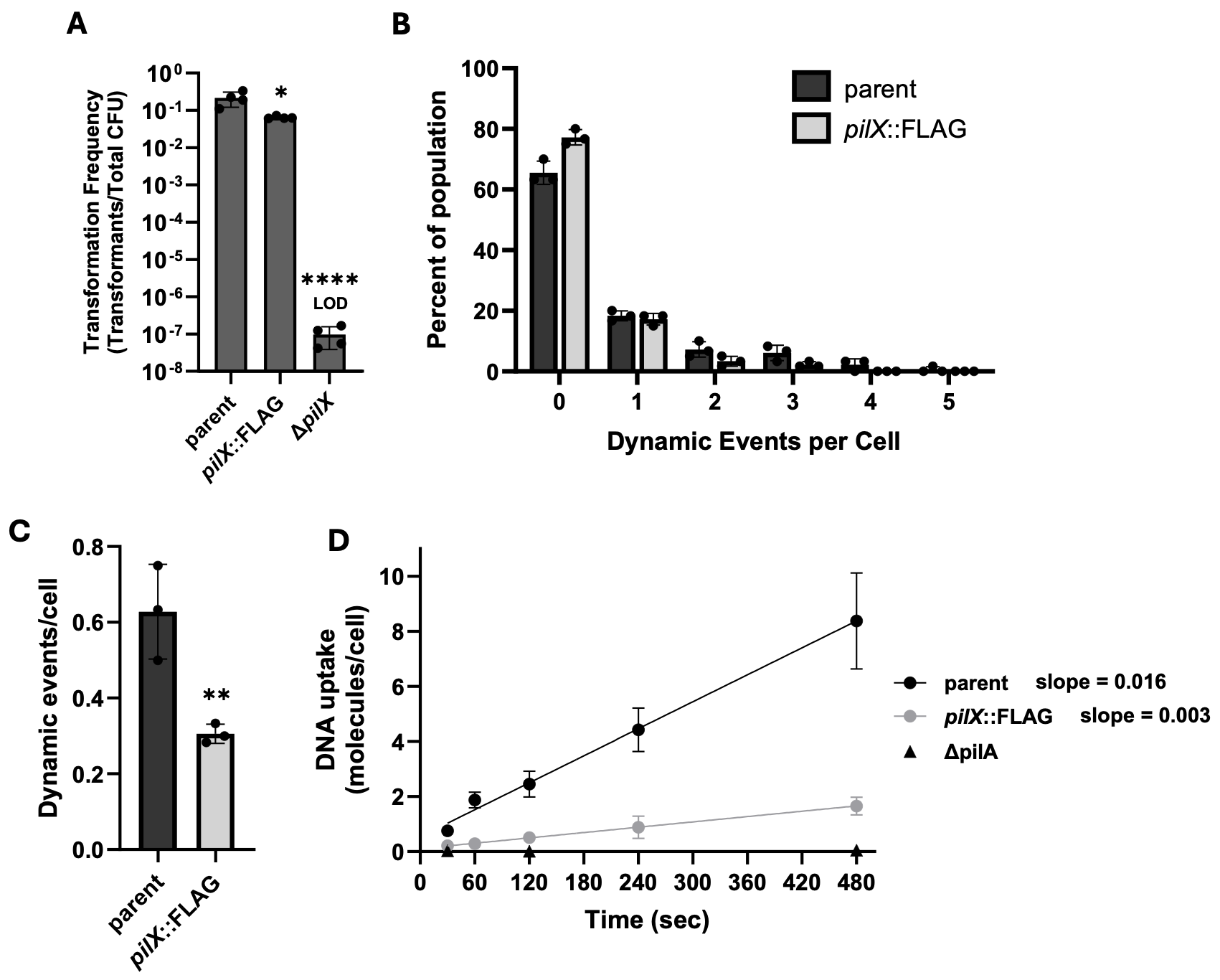

Supplement: S3 Fig — (A) Transformation assays of the indicated strains. (B-C) The frequency of T4aP dynamics of the indicated strains was quantified from timelapse imaging of AF488-mal labeled cells and the (B) distribution and (C) average T4aP dynamics per cell are plotted. Parent data shown here are identical to data shown in Fig 2B and 2C and are included here for ease of comparison. (D) DNA uptake into the periplasm of the indicated strains was kinetically monitored by qPCR. Parent and ΔpilA are identical to the data presented in Fig 2E and are included here for ease of comparison. Lines of best fit were determined by linear regression. The slope of each line is shown, which represents the rate of DNA uptake. Data is from at least 3 independent biological replicates and shown as the mean ± SD. Statistical comparisons were made in A by one-way ANOVA with Tukey’s multiple comparison test of the log-transformed data, and in C by unpaired Student’s t-test of the log-transformed data. * = p < 0.05, ** = p < 0.01, **** = p < 0.001. LOD = limit of detection. Statistical identifiers directly above bars represent comparisons to the parent. (TIFF) [file pgen.1012188.s003.tiff]

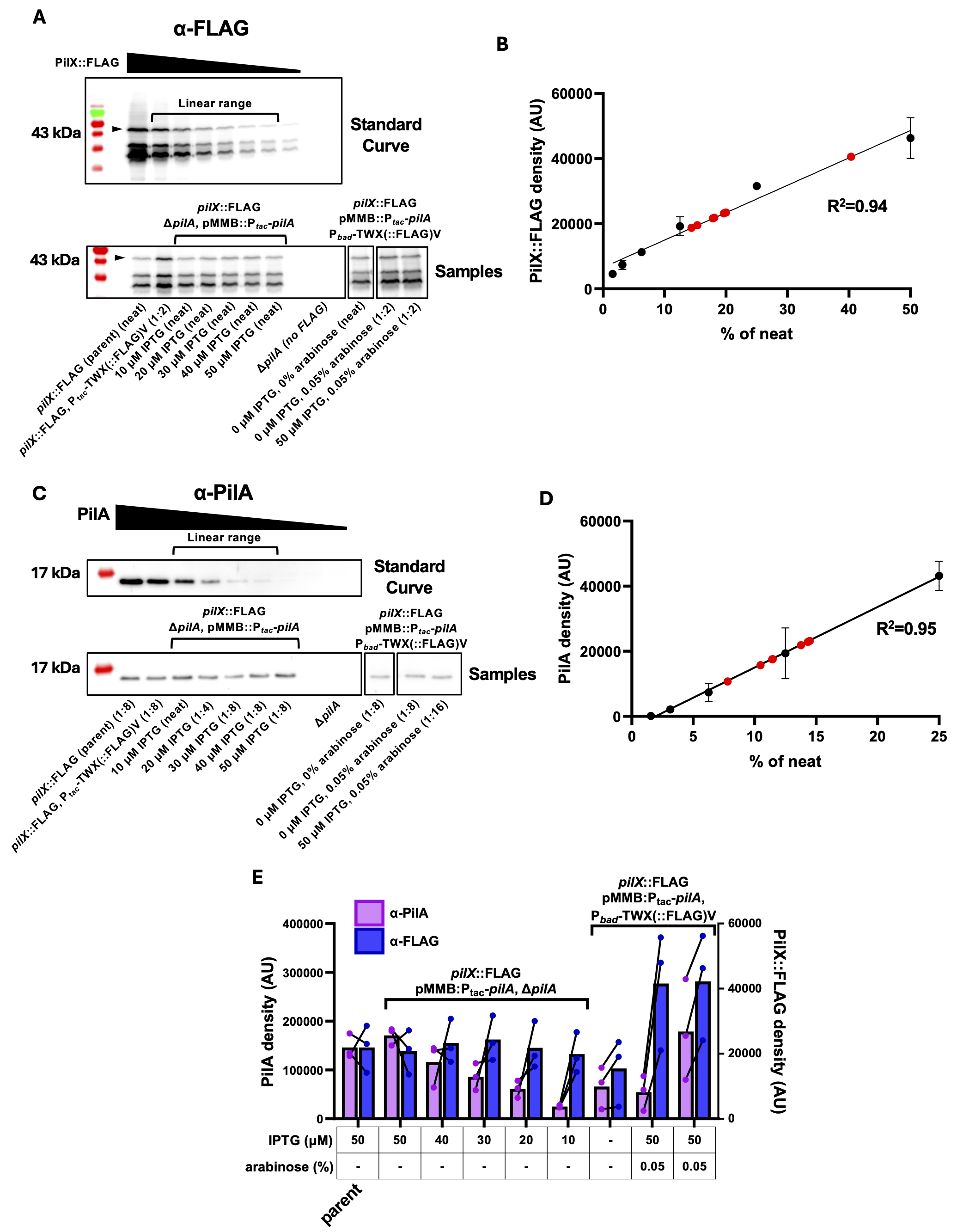

Supplement: S4 Fig — (A) Representative semi-quantitative western blots for detecting PilX::FLAG using α-FLAG primary antibodies. A serial 2-fold dilution series of PilX::FLAG (i.e., dilutions of a pilX::FLAG Ptac-TWX(::FLAG)V induced with 100 µM IPTG lysate) (top) was used to generate a standard curve to define the linear range of the assay. Experimental samples (bottom) from the indicated strains grown with the indicated concentration of inducer were diluted (dilution factor noted in parentheses) and blotted so that the signal would be within the linear range of the assay. The band that corresponds to full length PilX::FLAG (45 kDa) is demarcated with a black arrow, and the size of the closest ladder band is noted to the left of the blot. (B) Blots, as shown in A, were analyzed by densitometry and plotted. Black datapoints correspond to the standard curve (n = 3), and only data points within the linear range are shown. Linear regressions show the line of best fit for the standard curve and the R2 is shown on the plot. Red datapoints correspond to the densities of a subset of experimental samples (as depicted in A) to highlight that these data fall within the linear range of the assay. (C) Representative semi-quantitative western blots for detecting PilA via α-PilA antibodies. A 2-fold dilution series of PilA (i.e., dilutions of a ∆pilA pMMB::Ptac-pilA lysate) (top) was used to generate a standard curve to define the linear range of the assay. Experimental samples (bottom) from the indicated strains grown with the indicated concentration of inducer were diluted (dilution factor noted in parentheses) and blotted so that the signal would be within the linear range of the assay. (D) Blots, as shown in C, were analyzed by densitometry and plotted. Black datapoints correspond to the standard curve (n = 3), and only data points within the linear range are shown. Linear regressions show the line of best fit for the standard curve and the R2 is shown on the plot. Red datapoints correspond to t [file pgen.1012188.s004.tiff]

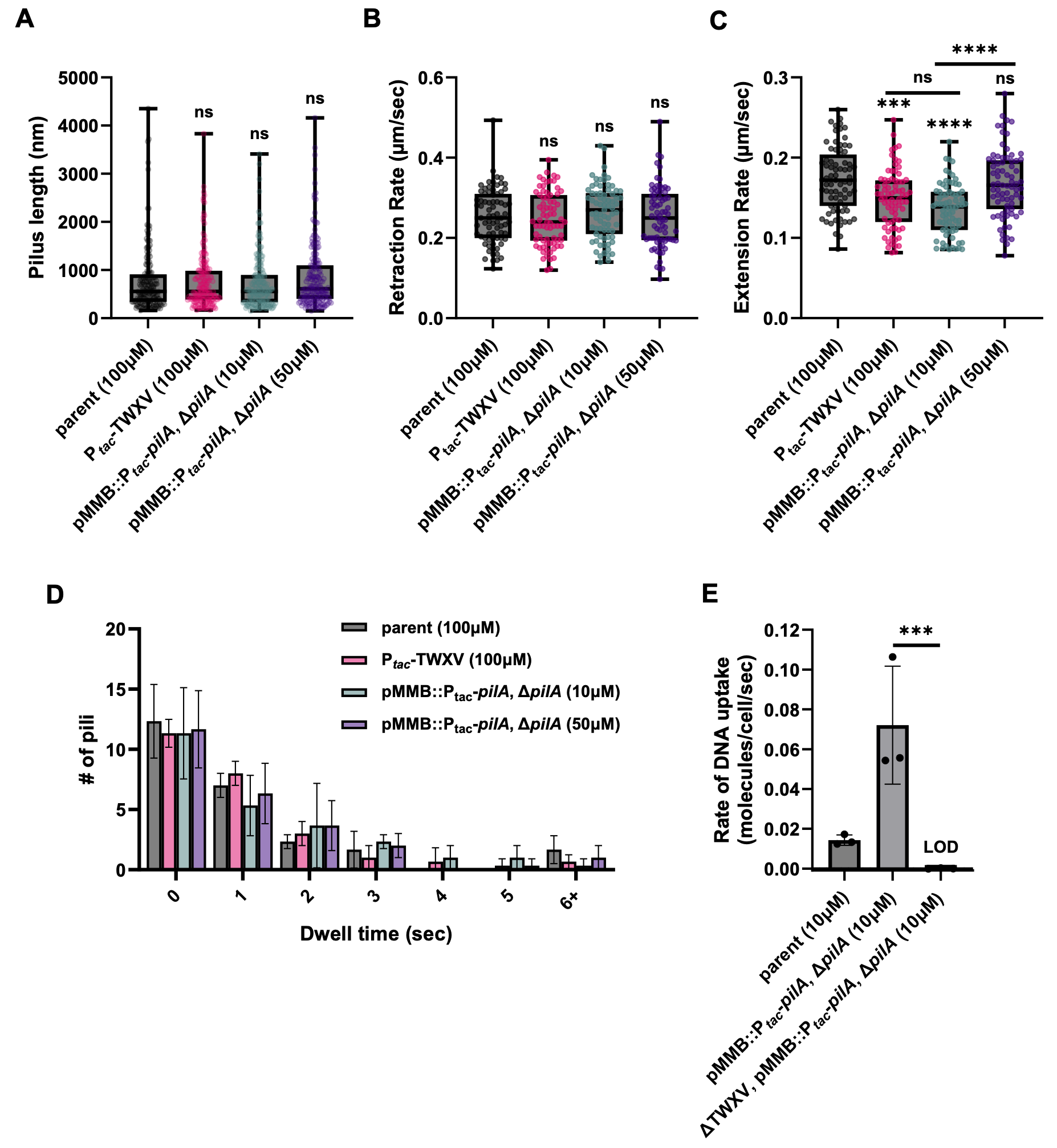

Supplement: S5 Fig — Timelapse imaging was performed on AF488-mal labeled cells of the indicated strains grown in the indicated concentration of IPTG and images were analyzed to determine the following properties of T4aP dynamics. (A) Maximum pilus lengths. (B) The rate of pilus retraction. (C) The rate of pilus extension. (D) The dwell time for pili. Dwell time is defined as the amount of time before an extended pilus begins to retract. (E) Rates of DNA uptake for the indicated strains derived from kinetic DNA uptake assays. All data are from 3 independent biological replicates and shown as the mean ± SD. Statistical comparisons were made by one-way ANOVA with Tukey’s multiple comparison test of the log-transformed data. ns = no significance, *** = p < 0.001, **** = p < 0.0001. Statistical identifiers directly above bars represent comparisons to the parent. (TIFF) [file pgen.1012188.s005.tiff]

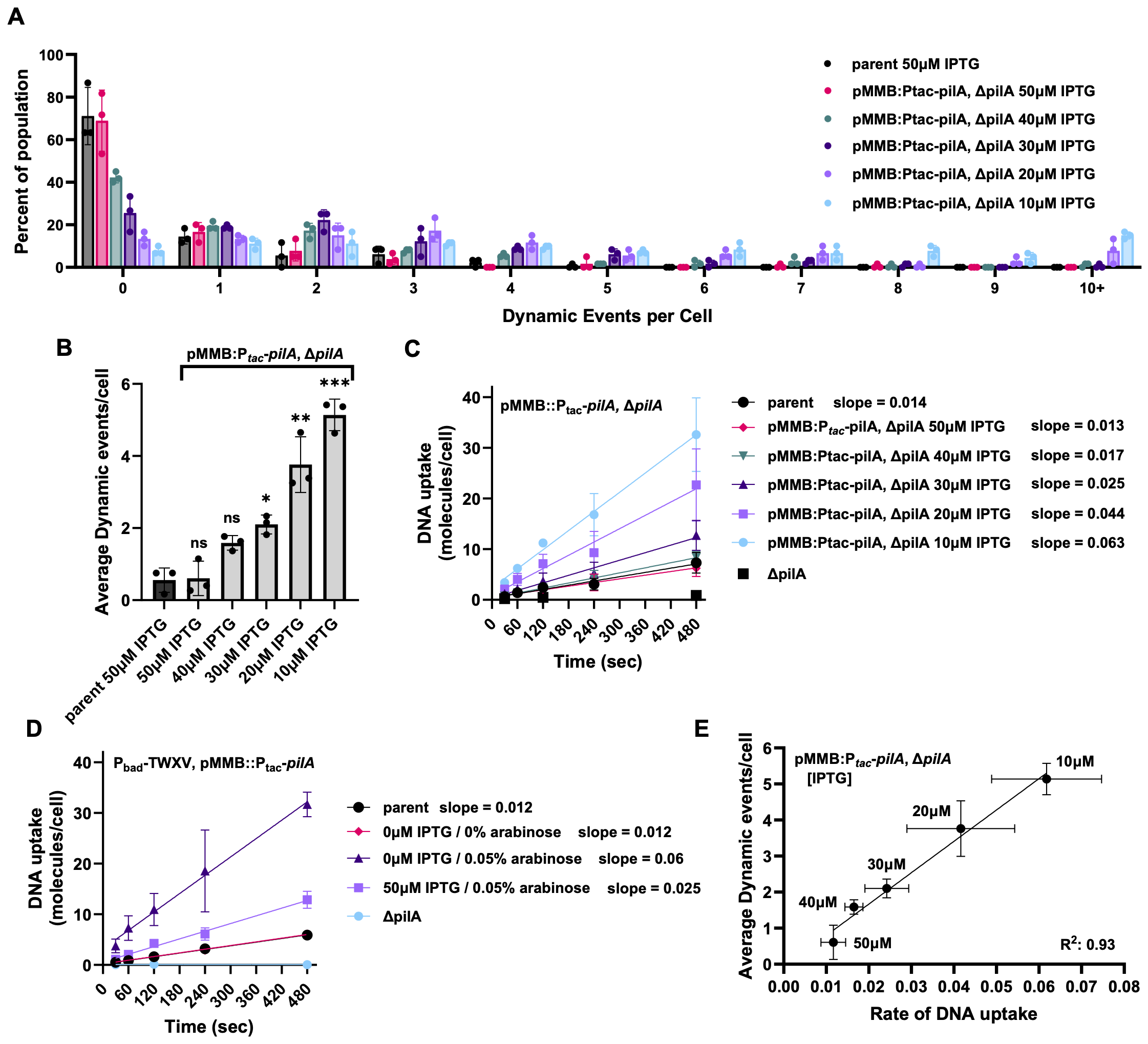

Supplement: S6 Fig — (A-B) Quantification of the frequency of T4aP dynamics from timelapse imaging of the indicated strains stained with AF488-mal with (A) distribution and (B) average dynamics per cell shown. (C-D) DNA uptake into the periplasm of the indicated strains grown in the indicated inducers was kinetically monitored by qPCR. Lines of best fit were determined by linear regression. The slope of each line is shown, which represents the rate of DNA uptake. The derived slope values serve as a functional correlate for the frequency of T4aP dynamics and are presented as bar graphs in Fig 3B. (E) The frequency of dynamic activity in B was plotted against the rate of DNA uptake in C at the indicated doses of IPTG to assess whether these properties are directly correlated. Linear regression analysis was performed and the R2 value is shown on the plot. All data are from 3 independent biological replicates and shown as the mean ± SD. Statistical comparisons were made by one-way ANOVA with Tukey’s multiple comparison test of the log-transformed data. ns = no significance, * = p < 0.05, ** = p < 0.01, *** = p < 0.001. Statistical identifiers directly above bars represent comparisons to the parent. (TIFF) [file pgen.1012188.s006.tiff]
