# Supplementary material for: The stoichiometry of minor-to-major pilins regulates the dynamic activity of the type IVa competence pilus in Vibrio cholerae
Source: PLoS Genet. 2026 Jun 4;22(6):e1012188. doi: 10.1371/journal.pgen.1012188 (PMC13249403; doi:10.1371/journal.pgen.1012188)
Supplement: S1 Table — (PDF) [file pgen.1012188.s007.pdf]

**Table S1** – *Strains used in this manuscript*

| Strain # | Genotype | Figures | Use in manuscript |
|---|---|---|---|
| NDC0547 / SAD4071 | Δ*pilT*::*Tm*$^{R}$, Δ*VC1807*::*Zeo*$^{R}$, P$_{const2}$-*tfoX*, Δ*luxO*, *lacZ*::*lacI*$^{q}$, *pilA*$^{S67C}$ | Fig. 1 | Δ*pilT* for surface piliation microscopy and quantification |
| NDC0585 / SAD4072 | Δ*pilQ*::*Tet*$^{R}$, Δ*pilT*::*Tm*$^{R}$, Δ*VC1807*::*Zeo*$^{R}$, P$_{const2}$-*tfoX*, Δ*luxO*, *lacZ*::*lacI*$^{q}$, *pilA*$^{S67C}$ | Fig. 1 | Δ*pilQ* control for surface piliation microscopy and quantification |
| NDC0575 / SAD4073 | Δ*fimT,* Δ*lacZ*::*Spec*$^{R}$, Δ*pilT*::*Tm*$^{R}$, Δ*VC1807*::*Zeo*$^{R}$, P$_{const2}$-*tfoX*, Δ*luxO*, *pilA*$^{S67C}$ | Fig. 1 | Δ*fimT* for surface piliation microscopy and quantification |
| NDC0758 / SAD4074 | Δ*pilW,* Δ*lacZ*::*Spec*$^{R}$, Δ*pilT*::*Tm*$^{R}$, Δ*VC1807*::*Kan*$^{R}$, P$_{const2}$-*tfoX*, Δ*luxO*, *pilA*$^{S67C}$ | Fig. 1 | Δ*pilW* for surface piliation microscopy and quantification |
| NDC0749 / SAD4075 | Δ*pilX,* Δ*lacZ*::*Spec*$^{R}$, Δ*pilT*::*Tm*$^{R}$, Δ*VC1807*::*Kan*$^{R}$, P$_{const2}$-*tfoX*, Δ*luxO*, *pilA*$^{S67C}$ | Fig. 1 | Δ*pilX* for surface piliation microscopy and quantification |
| NDC0751 / SAD4076 | Δ*pilV,* Δ*lacZ*::*Spec*$^{R}$, Δ*pilT*::*Tm*$^{R}$, Δ*VC1807*::*Kan*$^{R}$, P$_{const2}$-*tfoX*, Δ*luxO*, *pilA*$^{S67C}$ | Fig. 1 | Δ*pilV* for surface piliation microscopy and quantification |
| NDC0576 / SAD4077 | Δ*lacZ*::Tet$^{R}$-*lacI*-P$_{tac}$-*fimT*, Δ*fimT,* Δ*pilT*::*Tm*$^{R}$, Δ*VC1807*::*Zeo*$^{R}$, P$_{const2}$-*tfoX*, Δ*luxO*, *pilA*$^{S67C}$ | Fig. 1 | Δ*fimT* complement for surface piliation microscopy and quantification |
| NDC0752 / SAD4078 | Δ*lacZ*::Tet$^{R}$-*lacI*-P$_{tac}$-*PilW*, Δ*pilW,* Δ*pilT*::*Tm*$^{R}$, Δ*VC1807*::*Zeo*$^{R}$, P$_{const2}$-*tfoX*, Δ*luxO*, *pilA*$^{S67C}$ | Fig. 1 | Δ*pilW* complement for surface piliation microscopy and quantification |
| NDC0754 / SAD4079 | Δ*lacZ*::Tet$^{R}$-*lacI*-P$_{tac}$-*PilX*, Δ*pilX,* Δ*pilT*::*Tm*$^{R}$, Δ*VC1807*::*Zeo*$^{R}$, P$_{const2}$-*tfoX*, Δ*luxO*, *pilA*$^{S67C}$ | Fig. 1 | Δ*pilX* complement for surface piliation microscopy and quantification |
| NDC0755 / SAD4080 | Δ*lacZ*::Tet$^{R}$-*lacI*-P$_{tac}$-*PilV*, Δ*pilV,* Δ*pilT*::*Tm*$^{R}$, Δ*VC1807*::*Zeo*$^{R}$, P$_{const2}$-*tfoX*, Δ*luxO*, *pilA*$^{S67C}$ | Fig. 1 | Δ*pilV* complement for surface piliation microscopy and quantification |
| NDC0713 / SAD4081 | PilX G244::3xFLAG, Δ*ddmABC*::*Erm*$^{R}$, Δ*ddmDE*::*Kan*$^{R}$ Δ*VC1807*::*Cm*$^{R}$, P$_{const2}$-*tfoX*, Δ*luxO*, *lacZ*::*lacI*$^{q}$, *pilA*$^{S67C}$ | Fig. 2D, 3A, S3, S4 | parent for internally FLAG-tagged PilX (inserted after residue G244) for western blotting. |
| NDC0719 / SAD4082 | Δ*lacZ*::Tet$^{R}$-*lacI*-P$_{tac}$-*TWX(3xFLAG)V,* PilX G244::3xFLAG, Δ*ddmABC*::*Erm*$^{R}$, Δ*ddmDE*::*Kan*$^{R}$ Δ*VC1807*::*Cm*$^{R}$, P$_{const2}$-*tfoX*, Δ*luxO*, *pilA*$^{S67C}$ | Fig. 2D, S4 | Minor pilin overexpression strain for western blotting. The internal FLAG tag is present in both the native and ectopic copies of PilX. |
| NDC0761 / SAD4083 | Δ*pilA*, Δ*ddmABC*::*Erm*$^{R}$, Δ*ddmDE*::*Kan*$^{R}$ Δ*VC1807*::*Tm*$^{R}$, P$_{const2}$-*tfoX*, Δ*luxO*, *lacZ*::*lacI*$^{q}$, *pilA*$^{S67C}$ | Fig. 2DE, S4, S6 | Δ*pilA* and “NO FLAG” control for western blotting and DNA uptake assays |
| NDC0707 / SAD4084 | Δ*ddmABC*::*Erm*$^{R}$, Δ*ddmDE*::*Kan*$^{R}$ Δ*VC1807*::*Zeo*$^{R}$, P$_{const2}$-*tfoX*, Δ*luxO*, *lacZ*::*lacI*$^{q}$, *pilA*$^{S67C}$ | Fig. 1, 2A-C, 1EF, 3, S3, S5, S6 | Parent for measuring surface piliation, dynamic activity, DNA uptake, pilus length, pilus dwell time, and pilus extension/retraction rates |
| NDC0746 / SAD4085 | Δ*lacZ*::Tet$^{R}$-*lacI*-P$_{tac}$-*TWXV*, Δ*ddmABC*::*Erm*$^{R}$, Δ*ddmDE*::*Kan*$^{R}$ Δ*VC1807*::*Zeo*$^{R}$, P$_{const2}$-*tfoX*, Δ*luxO*, *pilA*$^{S67C}$ | Fig. 2A-C, 2E-F, 3B, S5 | Minor pilin overexpression strain for measuring dynamic activity, DNA uptake, pilus length, pilus dwell time, and pilus extension/retraction rates |

| | | | |
|---|---|---|---|
| NDC0763 / SAD4086 | pMMB::$P_{tac}$-*pilA Spec$^R$ Carb$^R$*, Δ*pilA*, PilX G244::3xFLAG, Δ*ddmABC*::*Erm$^R$*, Δ*ddmDE*::*Kan$^R$* Δ*VC1807*::*Cm$^R$*, $P_{const2}$-*tfoX*, Δ*luxO*, *lacZ*::*lacI$^q$*, *pilA$^{S67C}$* | Fig. 3A, S4 | Major pilin titratable expression strain with FLAG-tagged PilX for western blotting |
| NDC0730 / SAD4087 | pMMB::$P_{tac}$-*pilA Spec$^R$ Carb$^R$*, Δ*pilA*, Δ*ddmABC*::*Erm$^R$*, Δ*ddmDE*::*Kan$^R$* Δ*VC1807*::*Cm$^R$*, $P_{const2}$-*tfoX*, Δ*luxO*, *lacZ*::*lacI$^q$*, *pilA$^{S67C}$* | Fig. 3B-D, S5, S6 | Major pilin titratable expression strain for measuring dynamic activity, DNA uptake, pilus length, pilus dwell time, and pilus extension/retraction rates |
| NDC0815 / SAD4155 | Δ*TWXV::Spec$^R$*, Δ*pilT*::*Tm$^R$*, Δ*VC1807*::*Zeo$^R$*, $P_{const2}$-*tfoX*, Δ*luxO*, *lacZ*::*lacI$^q$*, *pilA$^{S67C}$* | Fig. 1 | Δ*TWXV* for surface piliation microscopy and quantification |
| NDC0820 / SAD4156 | Δ*lacZ*::Tet$^R$-*lacI*-$P_{tac}$-*TWXV*, Δ*TWXV::Spec$^R$*, Δ*pilT*::*Tm$^R$*, Δ*VC1807*::*Zeo$^R$*, $P_{const2}$-*tfoX*, Δ*luxO*, *pilA$^{S67C}$* | Fig. 1 | Δ*TWXV* complement for surface piliation microscopy and quantification |
| NDC0814 / SAD4157 | Δ*TWXV::Spec$^R$*, Δ*VC1807*::*Zeo$^R$*, $P_{const2}$-*tfoX*, Δ*luxO*, *lacZ*::*lacI$^q$*, *pilA$^{S67C}$* | Fig. 2A-C | Δ*TWXV* for measuring dynamic activity |
| NDC0812 / SAD4158 | pMMB::$P_{tac}$-*pilA Spec$^R$ Carb$^R$*, Δ*lacZ::Carb$^R$-araC-$P_{bad}$-TWX(::3xFLAG)V, pilX* G244::3xFLAG, Δ*ddmABC*::*Erm$^R$*, Δ*ddmDE*::*Kan$^R$* Δ*VC1807*::*Cm$^R$*, $P_{const2}$-*tfoX*, Δ*luxO*, *pilA$^{S67C}$* | Fig. 3A, 3CD, S4E | Minor and Major pilin overexpression strain for western blotting. The internal FLAG tag is present in both native and ectopic copies of the minor pilins |
| NDC0769 / SAD4159 | pMMB::$P_{tac}$-*pilA Spec$^R$ Carb$^R$*, Δ*lacZ::Carb$^R$-araC-$P_{bad}$-TWXV*, Δ*ddmABC*::*Erm$^R$*, Δ*ddmDE*::*Kan$^R$* Δ*VC1807*::*Cm$^R$*, $P_{const2}$-*tfoX*, Δ*luxO*, *pilA$^{S67C}$* | Fig. 3B-D, S6 | Minor and major pilin overexpression strain for DNA uptake |
| NDC0700 / SAD4160 | Δ*pilT*::*Tm$^R$*, Δ*epsM::Erm$^R$*, Δ*vesC::Cm$^R$*, Δ*VC1807*::*Zeo$^R$*, $P_{const2}$-*tfoX*, Δ*luxO*, *lacZ*::*lacI$^q$*, *pilA$^{S67C}$* | Fig. S1 | Δ*pilT* for T2SS cross-complementation surface piliation and quantification |
| NDC0704 / SAD4161 | Δ*pilQ::Tet$^R$*, Δ*pilT*::*Tm$^R$*, Δ*epsM::Erm$^R$*, Δ*vesC::Cm$^R$*, Δ*VC1807*::*Zeo$^R$*, $P_{const2}$-*tfoX*, Δ*luxO*, *lacZ*::*lacI$^q$*, *pilA$^{S67C}$* | Fig. S1 | Δ*pilQ* for T2SS cross-complementation surface piliation and quantification |
| NDC0694 / SAD4162 | Δ*epsHIJK::Kan$^R$*, Δ*pilT*::*Tm$^R$*, Δ*epsM::Erm$^R$*, Δ*vesC::Cm$^R$*, Δ*VC1807*::*Zeo$^R$*, $P_{const2}$-*tfoX*, Δ*luxO*, *lacZ*::*lacI$^q$*, *pilA$^{S67C}$* | Fig. S1 | Δ*epsHIJK* (T2SS minor pilins) for T2SS cross-complementation surface piliation and quantification |
| NDC0824 / SAD4163 | Δ*TWXV::Spec$^R$*, Δ*pilT*::*Tm$^R$*, Δ*epsM::Erm$^R$*, Δ*vesC::Cm$^R$*, Δ*VC1807*::*Zeo$^R$*, $P_{const2}$-*tfoX*, Δ*luxO*, *lacZ*::*lacI$^q$*, *pilA$^{S67C}$* | Fig. S1 | Δ*TWXV* for T2SS cross-complementation surface piliation and quantification |
| NDC0823 / SAD4164 | Δ*TWXV::Spec$^R$*, Δ*epsHIJK::Kan$^R$*, Δ*pilT*::*Tm$^R$*, Δ*epsM::Erm$^R$*, Δ*vesC::Cm$^R$*, Δ*VC1807*::*Zeo$^R$*, $P_{const2}$-*tfoX*, Δ*luxO*, *lacZ*::*lacI$^q$*, *pilA$^{S67C}$* | Fig. S1 | Δ*TWXV* Δ*epsHIK* for T2SS cross-complementation surface piliation and quantification |
| NDC0825 / SAD4165 | Δ*lacZ::Tet$^R$-lacI-$P_{tac}$-epsHIJK* Δ*TWXV::Spec$^R$*, Δ*pilT*::*Tm$^R$*, Δ*epsM::Erm$^R$*, Δ*vesC::Cm$^R$*, | Fig. S1 | Δ*TWXV epsHIJK* overexpression strain for T2SS cross- |

| | | | |
|---|---|---|---|
| | Δ*VC1807*::*Zeo*$^R$, $P_{const2}$-*tfoX*, Δ*luxO*, *pilA*$^{S67C}$ | | complementation surface piliation and quantification |
| NDC0818 / SAD4166 | Δ*TWXV::Spec*$^R$, pMMB::$P_{tac}$-*pilA Spec*$^R$ *Carb*$^R$, Δ*pilA*, Δ*ddmABC*::*Erm*$^R$, Δ*ddmDE*::*Kan*$^R$ Δ*VC1807*::*Cm*$^R$, $P_{const2}$-*tfoX*, Δ*luxO*, *lacZ*::*lacI*$^q$, *pilA*$^{S67C}$ | Fig. S5E | Δ*TWXV* pMMB::$P_{tac}$-*pilA* Δ*pilA* for DNA uptake assays |
| NDC0748 / SAD4167 | Δ*pilX*, Δ*lacZ*::*Spec*$^R$, Δ*VC1807*::*Kan*$^R$, $P_{const2}$-*tfoX*, Δ*luxO*, *pilA*$^{S67C}$ | Fig. S3A | Δ*pilX* for transformation assays |
