# Supplementary material for: The stoichiometry of minor-to-major pilins regulates the dynamic activity of the type IVa competence pilus in Vibrio cholerae
Source: PLoS Genet. 2026 Jun 4;22(6):e1012188. doi: 10.1371/journal.pgen.1012188 (PMC13249403; doi:10.1371/journal.pgen.1012188)
Supplement: S2 Table — (PDF) [file pgen.1012188.s008.pdf]

Table S2 – *Primers used in this study*

Primer #	Sequence	Use
DOG0400	ACTTCTGGCTGAAGGTCAATTTTC	Δ*pilT* F1
DOG0401	gtcgacggatccccggaatCATTTAAATTCCTTAATAAAGTCTGGC	Δ*pilT* R1
DOG0402	gaagcagctccagcctacaTAGGTAGGTAAAGACAGATGGAG	Δ*pilT* F2
DOG0403	TCACGTGTTCGGCCAAAATC	Δ*pilT* R2
DOG0404	TTCTGCTTGCCTTGCGTC	Δ*pilT* detect
DOG0582	ACATCTGGCAGATCTGATTTCAC	Δ*pilQ* F1
DOG0583	gtcgacggatccccggaatCAACCGATTATTTTAAAGCCAGCTTG	Δ*pilQ* R1
DOG0584	gaagcagctccagcctacaTAACTTAGCGTGTGCGTAACAAG	Δ*pilQ* F2
DOG0585	TTGTGTTTGCTCTAACGTTTGC	Δ*pilQ* R2
DOG0586	ACAAGAGTTGCTGGACATACG	Δ*pilQ* detect
DOG0549	AACGACATTACTGACGTTATCCTAG	Δ*fimT* F1/ΔTWXV F1
DOG0550	gtcgacggatccccggaatATGCATTTCCCAAAATCCTTTGC	Δ*fimT* R1
DOG0551	gaagcagctccagcctacaGCTGGACGAATCAAAGTTTGC	Δ*fimT* F2
DOG0552	AATGATACCGCCCAGAGGAATC	Δ*fimT* R2
DOG0553	ATCACACCGACAGCACCA	Δ*fimT* detect
DOG0554	gtcgacggatccccggaatTTGCTGGTTAGCACTTTTCAAAGC	Δ*pilW* R1
DOG0555	gaagcagctccagcctacaGCTATCTCATTACAGGTTCAGC	Δ*pilW* F2
DOG0556	TCGAACATAAATATCAGAAGCTGA	Δ*pilW* detect
DOG0557	TGGTGACTGAGAACATGTCAAAAC	Δ*pilX* F1
DOG0558	gtcgacggatccccggaatAATCATTATTTCCAATTCCTTTGCTG	Δ*pilX* R1
DOG0559	gaagcagctccagcctacaGATGTGATCGATCATATTGCGAG	Δ*pilX* F2
DOG0560	TCATCGTGCATTTGTCCAGTTAAG	Δ*pilX* R2/ΔTWXV R2
DOG0561	ATGAGTTTGCACTTATCAGCG	Δ*pilX* detect
DOG0562	gtcgacggatccccggaatACCAGATTGCTTATTGCGC	Δ*pilV* R1
DOG0563	gaagcagctccagcctacaTGAACGAGTCCTTCTTGCC	Δ*pilV* F2
DOG0564	TAGTGATAAAGCCTGAAGAGTCAG	Δ*pilV* detect
BBC1944	caatttcacacaggatcccgggAGGAGGTTGGGAAATGCATCGCGGCT TTAC	P_{tac}-*fimT* F
BBC1945	tgtaggctggagctgcttcGGTTAGCACTTTTCAAAGCCATA	P_{tac}-*fimT* R
BBC1946	caatttcacacaggatcccgggAGGAGGTCAAAATATGGCTTTGAAAA GTGC	P_{tac}-*pilW* F
BBC1947	tgtaggctggagctgcttcTTATTTCCAATTCCTTTGCTGAACC	P_{tac}-*pilW* R
BBC1948	caatttcacacaggatcccgggAGGAGGTGAAATAATGATTCAATATCA AAAAGGTGTC	P_{tac}-*pilX* F
BBC1949	tgtaggctggagctgcttcTTATTGCGCATTCCAGCTTC	P_{tac}-*pilX* R
BBC1950	caatttcacacaggatcccgggAGGAGGTGCTGGAATGCGCAATAAGC	P_{tac}-*pilV* F
BBC1951	tgtaggctggagctgcttcTCAGTCAAATTCGCTGTATTTAGAG	P_{tac}-*pilV* R
DOG0548	AAGAGTTTGAAGAGTCTTGTGGC	*pilX* G244::3xFLAG F1
NCP0229	tccaccacttccacctgcACCATTCGTATTTTCTGGAGC	*pilX* G244::3xFLAG R1
NCP0230	gcaggtggagcaggtggaCAACCTAGTATTGTTCCTGATTG	*pilX* G244::3xFLAG F2
BBC1874	GAGACGCTGACAAAATCACAC	*pilX* G244::3xFLAG R2
BBC993	ttgattataaggatgacgatgac	*pilX* G244::3xFLAG detect F
NCP0239	CTCGCAATATGATCGATCACATC	*pilX* G244::3xFLAG detect R

NCP0486	caatttcacacaggatcccgggTTTGCAAAGGATTTTGGGAAATG	P_{tac}/P_{bad}-*TWXV* F
DOG0565	AGATTGGTCTATTTTATGGCTCAAG	Δ*pilA* F1
DOG0566	gtcgacggatccccggaatCATATGCCTTGCTACACAAGG	Δ*pilA* R1
DOG0567	gaagcagctccagcctacaCCAAAAGGCTGTACTGCAGG	Δ*pilA* F2
DOG0568	AGGGTCAGTTTACCCTCTGG	Δ*pilA* R2
DOG0569	TACTTAACTCAAGCAGCGCC	Δ*pilA* detect
BBC4286	AGATATAGCGAATGACCGTTTGC	Δ*ddmABC* F1
BBC4287	gtcgacggatccccggaatCATTCAACCTCTTATGAAATCATACC	Δ*ddmABC* R1
BBC4288	gaagcagctccagcctacaCCGCTGGATTGGCTATAAAGC	Δ*ddmABC* F2
BBC4289	AAGATAGCGCAGAGCTTCAG	Δ*ddmABC* R2
BBC4290	AGCTTACTTGACCCATTGTCTG	Δ*ddmABC* detect
BBC4212	ATGGAACCGAAACGTGATCAG	Δ*ddmDE* F1
BBC4213	gtcgacggatccccggaatGAACATGTCGAATAACTAGACAGG	Δ*ddmDE* R1
BBC4214	gaagcagctccagcctacaTGATAGCAGACTAAACCACGG	Δ*ddmDE* F2
BBC4215	TCAGCAATTAAGCCTTCCG	Δ*ddmDE* R2
BBC4216	GTTTGTTATGTACTGAGTGGTCC	Δ*ddmDE* detect
JCP313	caatttcacacaggatcccgggAGGAGGTGAAATTATGAAAGCG	P_{tac}-*pilA* F
BBC1884	tgtaggctggagctgcttcTTAGTTAATTGTTGCACCTGC	P_{tac}-*pilA* R
BBC2822	aatttcacacaggaaacagaattcgagctcTTGTTTCGGCGTGGGTATGGT G	To clone P_{tac}-*pilA* onto pMMB F
BBC2823	caggtcgactctagaggatccccgggtacCtgtaggctggagctgcttc	To clone P_{tac}-*pilA* onto pMMB R
BBC2824	gagctcgaattctgtttcctgtgtgaaattTTTGCCAGAACCGTTATGATGTC	pMMB F
BBC2357	GGTACCCGGGGATCCTCTAGAGTCGACCTGCAGGCATGCAA GCTTGGC	pMMB R
NCP0475	ACGGACTTGGTACTGGAAG	Δ*epsM* F1
NCP0476	gtcgacggatccccggaatTTCTTTCATCATTTCTCCTTACTTGG	Δ*epsM* R1
NCP0477	gaagcagctccagcctacaTGATATGAAGCGTGCTGTTG	Δ*epsM* F2
NCP0478	GTAGAATGGTGAGCAATAACC	Δ*epsM* R2
NCP0479	CTCTTAGTTGTGTGCTGCTAC	Δ*epsM* detect
BBC4281	TGCTCAAACTGCTCTCGAG	Δ*vesC* F1
BBC4282	gtcgacggatccccggaatGTTCATAGATACCTCTGATAACTCC	Δ*vesC* R1
BBC4283	gaagcagctccagcctacaCGCGTCTGATCTATCGATAGCC	Δ*vesC* F2
BBC4284	GCCATGAAGGCACTCTCTTC	Δ*vesC* R2
BBC4285	CTTGCAGCTCAAGGCATTAC	Δ*vesC* detect
NCP0490	gtcgacggatccccggaatACCCGTTATAGAAGGCAATCC	ΔTWXV R1
BBC1873	gaagcagctccagcctacaTGAACGAGTCCTTCTTGCC	ΔTWXV F2
BBC2060	GGTGATCTCTCCAGCTGAG	Δ*epsHIJK* F1
NDC0020	gtcgacggatccccggaatCATAAGGTTCCGTCAGTTACCG	Δ*epsHIJK* R1
NDC0024	gaagcagctccagcctacaAAACTGCGACGGTGATACG	Δ*epsHIJK* F2
NDC0022	GCTCGTGGAAAATAACCATCC	Δ*epsHIJK* R2
NCP0480	caatttcacacaggatcccgggGCGGTAACTGACGGAACCTTATG	P_{tac}-HIJK F
NCP0481	tgtaggctggagctgcttcCTACTCAGTCGAACGGTC	P_{tac}-HIJK R
